# Supplementary material for: An operant-based touchscreen morph discrimination task does not detect age-related mnemonic similarity deficits in rats
Source: Oxf Open Neurosci. 2026 Oct 1;5:kvag015. doi: 10.1093/oons/kvag015 (PMC13647288; doi:10.1093/oons/kvag015)
Supplement: Supplementary file 1 [file supplementary_meterial_v1_kvag015.docx]

**Decision Letter (OXFNSC-2026-017)**

**08-Jun-2026**

Dear Dr. Burke,

Manuscript ID OXFNSC-2026-017 entitled "An Operant-based Touchscreen Morph Discrimination Task Does Not Detect Age-related Mnemonic Similarity Deficits in Rats" which you submitted to the Oxford Open Neuroscience, has been reviewed. The comments of the reviewers are included at the bottom of this letter.

Please note that this journal operates with transparent peer review. This means that if your submission is accepted for publication, the full peer review history of your article will publish online alongside your article. This includes reviewer comments, editor decision letters, and your author responses.

The reviewers were favorably inclined but also suggest some major revisions to your manuscript. Therefore, I invite you to respond to the reviewers' comments and revise your manuscript within the next 60 days from today date.

To revise your manuscript, log into https://mc.manuscriptcentral.com/oxfnsc and enter your Author Centre, where you will find your manuscript title listed under "Manuscripts with Decisions."  Under "Actions," click on "Create a Revision."  Your manuscript number has been appended to denote a revision.

You may also click the below link to start the revision process (or continue the process if you have already started your revision) for your manuscript. If you use the below link you will not be required to login to ScholarOne Manuscripts.

*** PLEASE NOTE: This is a two-step process. After clicking on the link, you will be directed to a webpage to confirm. ***

https://mc.manuscriptcentral.com/oxfnsc?URL_MASK=7d1bd4aec90a44b68fab74ff2a0fbc22

You will be unable to make your revisions on the originally submitted version of the manuscript.  Instead, revise your manuscript using a word processing program and save it on your computer.  Please also highlight the changes to your manuscript within the document by using the track changes mode in MS Word or by using bold or colored text.

<strong>REMINDER:</strong> if you have not already done so, when submitting your revised manuscript, please include a short alternative-text description under the figure legend for each figure. Alt-text is required to ensure the accessibility of our publications. Guidance is available on the journal’s Author Guidelines webpage and at <a href="https://academic.oup.com/pages/for-authors/journals/preparing-and-submitting-your-manuscript" target="_new">https://academic.oup.com/pages/for-authors/journals/preparing-and-submitting-your-manuscript</a>

Once the revised manuscript is prepared, you can upload it and submit it through your Author Centre.

When submitting your revised manuscript, you will be able to respond to the comments made by the reviewer(s) in the space provided.  You can use this space to document any changes you make to the original manuscript.  In order to expedite the processing of the revised manuscript, please be as specific as possible in your response to the reviewer(s).

IMPORTANT: Your original files are available to you when you upload your revised manuscript.  Please delete any redundant files before completing the submission.

Because we are trying to facilitate timely publication of manuscripts submitted to the Oxford Open Neuroscience, your revised manuscript should be uploaded within the next 60 days from today date.  If it is not possible for you to submit your revision in a reasonable amount of time, we may have to consider your paper as a new submission.

Once again, thank you for submitting your manuscript to the Oxford Open Neuroscience and I look forward to receiving your revision.

Sincerely,
Prof. Alicia Izquierdo
Senior Editor, Oxford Open Neuroscience
aizquie@psych.ucla.edu


Reviewer: 1

Comments to the Author
I enjoyed reading this manuscript and think it addresses an important and timely question. The importance of cross-species alignment in cognitive tasks cannot be overstated, especially for tasks like the Mnemonic Similarity Task that are used to link human behavior, aging, and hippocampal-cortical mechanisms. Developing a touchscreen-based rodent version using 2D stimuli is a valuable goal because it could improve automation, standardization, and translational comparability with human testing.

The main result is clear and useful. Rats showed systematically reduced performance as image similarity increased, demonstrating that the morph manipulation successfully modulated discrimination difficulty. However, young and aged rats did not differ across overlap conditions, and perirhinal cortical Arc expression did not reliably differ by age or overlap condition. This is an important finding: the task appears sensitive to visual similarity, but it does not capture the age-related mnemonic discrimination deficits previously observed in 3D object-based rodent MST paradigms.

Major points
1. The distinction between platform alignment and construct alignment is central.
One of the most interesting aspects of the manuscript is that the task improves surface-level alignment with the human MST by using 2D image stimuli and an automated touchscreen platform. However, the findings suggest that this may not be sufficient to preserve the same cognitive construct. This is not a criticism of the study so much as an important translational note. A task can look more similar across species while still being solved using different cognitive processes. It begs the question on whether we should be focusing on aligning tasks based on platform or leverage species-specific advantages. For example, given rodents strong sense of smell I always wonder if we should be focusing on olfactory memory in this species. Was always impressed with Howard Eichenbaum’s olfactory behavior paradigms. Of course, there are issues with this too. While the hippocampus is often thought of as domain-agnostic, other MTL regions may not be.
2. Repeated stimulus exposure may shift the task away from mnemonic discrimination.
In the human MST, participants encode many objects and later discriminate repeated targets from similar lures. In the current task, rats repeatedly encounter a small set of images along a single bulldog-owl morph continuum. Each morph session included repeated presentations of the same overlap conditions, and animals completed multiple morph sessions. I would hypothesize that this would enable rats to make stable stimulus associations for specific images rather than relying on trial-unique mnemonic lure discrimination. This may result in putting stronger demands on perceptual systems. Obviously the MST and all related tasks rely on perception and mnemonic discrimination, but the degrees to which these systems drive performance likely differ by task.
3. Two-alternative forced-choice tasks allow strategy differences.
The 2AFC design is practical and appropriate for touchscreen work, but it introduces interpretive ambiguity. Humans often use explicit mnemonic strategies on 2AFC tasks, whereas nonhuman animals may default to reinforced stimulus-response or habit-based strategies after repeated training. A good example of this is how hippo lesions impair visual discrimination while they don’t in monkeys. There is also work in primates on tasks such as transverse patterning, where strategy use can differ across species resulting in differences in age effects. There can, of course, still be age-related deficits on 2AFC as has been shown by the authors previously in macaques. However, it is still worth thinking about different strategies. The side-bias analyses are helpful, but they do not fully rule out more subtle strategy differences. This isn’t a flaw by any means, but I think its something worth discussing. Not sure if there are analyses that can be done to try to disentangle this? Maybe looking at learning curves on an individual level to see if there is gradual learning?
4. The lack of learning across days is helpful, but trial-type-specific learning could be clarified.
The authors report no significant main effect of testing day, suggesting that performance did not generally improve across the three morph testing days. However, it would be useful to clarify whether there was a day × trial type interaction, since this would address whether animals improved specifically on harder morph conditions over time. This matters because learning on specific trial types could further support the idea that animals were acquiring stimulus-response mappings.
5. The perirhinal Arc analysis is interesting, but hippocampus seems essential for an MST-framed task.
The focus on perirhinal cortex is justified given its role in object and feature processing. However, given the framing around mnemonic similarity and the broader MST literature, I was surprised that hippocampal regions were not examined, particularly dentate gyrus/CA3. A fully functioning perirhinal cortex with dysfunctional hippocampal pattern separation circuitry could still produce mnemonic discrimination impairments under prevailing models. Therefore, the null perirhinal Arc finding should be interpreted cautiously.
6. Strategy-related circuitry could be informative in future work.
Given the possibility that animals may solve the task using rule-based, procedural, or habit-based strategies, future neural analyses could also examine prefrontal cortex and striatal regions. This would help determine whether touchscreen morph performance depends on mnemonic discrimination circuitry or on alternative decision/habit systems.
Overall assessment
Overall, I think this is a strong and valuable manuscript. The negative result is important because it shows that touchscreen-based 2D morph discrimination can measure sensitivity to visual similarity in rats, but may not reproduce the age-related mnemonic deficits observed in more naturalistic 3D object-based paradigms. The paper’s broader contribution is that it highlights a key challenge for translational cognitive neuroscience: cross-species alignment is essential, but alignment of testing platform does not guarantee alignment of cognitive mechanism. Hopefully this will spark others to realize how important cross-species work is and why we need to do it!

Reviewer: 2

Comments to the Author
In An Operant-based Touchscreen Morph Discrimination Task Does Not Detect Age-related Mnemonic Similarity Deficits in Rats, the authors present a compelling set of experiments highlighting that while their novel touchscreen MST task shows expected decrements in performance as object similarity increases, it does not show the expected age-related decline seen in humans or in other rat versions of the task. Reporting on unexpected and important null results is important and this paper does that in a very clear and considered way.

I have mostly minor concerns and considerations for the authors to address in order for me to recommend this for publication.

Main concerns/considerations/suggestions:
- While the Arc experiment is interesting, I’m unsure how much value it adds to the paper.
1) Firstly, I’m not sure there is enough initial evidence to hypothesize that there would be differences in activity levels of the PRC with different levels of overlap (which is covered in the Discussion). So the lack of effect here is not the same as the lack of effect in the behavioural data where previous results have found a difference, in that case with age.
2) The sample size for this experiment is notably quite low, particularly in the aged group. Touchscreen experiments ideally have an n of 16 per group for good statistical power. Here, there are groups as low as 5, which is then divided by sex. In the very least, justification for this low sample size is needed. It should also be considered whether the statistical tests used are valid at such low sample sizes. I wonder if this experiment is better suited for supplemental information as “exploratory analyses” since I don’t think strong conclusions can be drawn from the low sample size.
3) Another thing to consider with the Arc experiment is the number of factors going into the main model and whether it would make better scientific or statistical sense to parcellate some of these tests. Particularly the LMM described on pp18-19 has 5 factors, but do the regions and layers need to be directly compared? Or is the more interesting and important analysis whether there are differences within regions? I also wonder if whether analyzing across sex when there are then some groups with ns of 2 or 3 is useful or appropriate or whether for these analyses it would make better statistical sense to collapse across sex.
In all, I question whether the Arc experiment should be removed from the paper, moved to a supplement, or reworked with these considerations in mind.
- While there is some discussion on whether the testing modality (touchscreen vs 3D objects in an arena) influences the outcomes in the MST here vs in previous studies, I think this needs expanded upon. Previous studies have also detected differences in hand-testing vs touchscreen testing (e.g. https://doi.org/10.1523/ENEURO.0120-24.2024) and attributed them to different levels of the cue-context relationship. Touchscreens present the stimuli in a context much more void of “extra-maze” information than tests in standard arenas. Since the results here do not align with previous conventional testing results, the difference likely lies in the context or the stimuli themselves.
- Speaking of the stimuli, I believe more discussion is needed on the aspect of what features might be attended to by the rats and how that may affect performance. I was struck by the choice to use images with faces as these contain features, such as eyes, that are naturally more focussed upon than other features. In the end, it is unclear whether the aged mice did better than expected or the young mice did worse; it could be that the facial features in the images reduced performance across the board. Discussion on why these ethologically-relevant images were chosen is needed, and perhaps reasons why images from the recent Stark et al MST which are known to work in humans were not used.

Minor concerns:
- Spelling and grammar need checked throughout. A few errors I caught:
p2 line 19: “are” is missing after “that”
p3 line 23 or 24: “unilateral knife cute”; I assume “cut”
p4 line 11: “yyoung” should be “young”
p10 lines 41-42: “Mopher” should be “Morpher”
p11 line 49 or 50: “To determine is the perirhinal cortex…”; not sure if that should be “if” or something else
- Page 7 and some figures mention the number of days required to reach criterion, but it has not been said how many touchscreen sessions occurred per day. More common notation for this is “Number of Sessions” as it is more specific.
- In Figure 3, the S+ and S- are not mentioned in the caption.
- On p9 the abbreviation OCSC is first introduced, and then OCSCT is immediately used, followed by “OCSC task”. If the T in OCSCT is for task, there should be consistency throughout and either only OCSCT or OCSC task be used
- I’m very curious as to what happens with the data if you do not collapse the overlaps together (e.g. averaging 25% overlap that is mostly dog with that which is mostly owl). Do rats perform any better with one of the stimuli than the other? This might be more my curiosity than a revision necessary in the manuscript!
- A lot of the data appear to violate normality assumptions for ANOVAs. Has this been checked and have alternative tests been used or considered?

**Author's Response to Decision Letter for (OXFNSC-2026-017)**

**21-Aug-2026**

Response to Reviewers
Manuscript OXFNSC-2026-017
"An Operant-based Touchscreen Morph Discrimination Task Does Not Detect Age-related Mnemonic Similarity Deficits in Rats"

We thank the reviewers for their careful reading of our manuscript and for their constructive and detailed feedback. We were glad that both reviewers found the central question and the negative result compelling, and we have used their comments to substantially strengthen the Discussion, correct several errors, and improve clarity and consistency throughout. Below we respond point-by-point with reviewer comments reproduced in quotes, followed by our response. Page numbers refer to the attached revised manuscript, in which all changes are indicated with blue font.

REVIEWER 1
We are thrilled that this reviewer enjoyed reading the paper and thank them for their insightful and helpful critique. Moreover, highlighting the translational importance of the platform-alignment/construct-alignment distinction has helped to improve and sharpen the manuscript. We thank them for their overall assessment that, “Overall, I think this is a strong and valuable manuscript. The negative result is important because it shows that touchscreen-based 2D morph discrimination can measure sensitivity to visual similarity in rats, but may not reproduce the age-related mnemonic deficits observed in more naturalistic 3D object-based paradigms. The paper’s broader contribution is that it highlights a key challenge for translational cognitive neuroscience: cross-species alignment is essential, but alignment of testing platform does not guarantee alignment of cognitive mechanism. Hopefully this will spark others to realize how important cross-species work is and why we need to do it!”

Major point 1:
" The distinction between platform alignment and construct alignment is central. One of the most interesting aspects of the manuscript is that the task improves surface-level alignment with the human MST by using 2D image stimuli and an automated touchscreen platform. However, the findings suggest that this may not be sufficient to preserve the same cognitive construct. This is not a criticism of the study so much as an important translational note. A task can look more similar across species while still being solved using different cognitive processes. It begs the question on whether we should be focusing on aligning tasks based on platform or leverage species-specific advantages. For example, given rodents strong sense of smell I always wonder if we should be focusing on olfactory memory in this species. Was always impressed with Howard Eichenbaum’s olfactory behavior paradigms. Of course, there are issues with this too. While the hippocampus is often thought of as domain-agnostic, other MTL regions may not be."
Response: We agree this is a central and clarifying framing for our findings, and we thank the reviewer for articulating it so clearly. We have added a new closing paragraph to the Discussion (pages 27-28) that explicitly adopts this platform-alignment versus construct-alignment framing and raises the possibility that leveraging species-typical sensory strengths (for example, olfactory-based paradigms; Yoder et al. (2014; 2017), already cited) may, in some cases, better preserve the underlying cognitive construct than matching testing platforms across species.

Major point 2:
" Repeated stimulus exposure may shift the task away from mnemonic discrimination. In the human MST, participants encode many objects and later discriminate repeated targets from similar lures. In the current task, rats repeatedly encounter a small set of images along a single bulldog-owl morph continuum. Each morph session included repeated presentations of the same overlap conditions, and animals completed multiple morph sessions. I would hypothesize that this would enable rats to make stable stimulus associations for specific images rather than relying on trial-unique mnemonic lure discrimination. This may result in putting stronger demands on perceptual systems. Obviously, the MST and all related tasks rely on perception and mnemonic discrimination, but the degrees to which these systems drive performance likely differ by task."
Response: We agree this is an important point, and likely we underappreciated the difference between the OCSC task used here and the human MST when we initially designed these experiments. We have added a new paragraph to the Discussion (page 25) that explicitly addresses the non-trial-unique, repeated-exposure design of our task, contrasts it with trial-unique paradigms such as the human MST and the rodent TUNL task (Talpos et al., 2010; Attalla et al., 2024; Zequeira et al., 2025, newly cited), which both detect differences between young and older groups. We now note that this could have allowed rats to solve the task via stable stimulus-response associations or habit-like responding (Packard and Knowlton, 2002, newly cited).

Major point 3:
"Two-alternative forced-choice tasks allow strategy differences. The 2AFC design is practical and appropriate for touchscreen work, but it introduces interpretive ambiguity. Humans often use explicit mnemonic strategies on 2AFC tasks, whereas nonhuman animals may default to reinforced stimulus-response or habit-based strategies after repeated training. A good example of this is how hippo lesions impair visual discrimination while they don’t in monkeys. There is also work in primates on tasks such as transverse patterning, where strategy use can differ across species resulting in differences in age effects. There can, of course, still be age-related deficits on 2AFC as has been shown by the authors previously in macaques. However, it is still worth thinking about different strategies. The side-bias analyses are helpful, but they do not fully rule out more subtle strategy differences. This isn’t a flaw by any means, but I think its something worth discussing. Not sure if there are analyses that can be done to try to disentangle this? Maybe looking at learning curves on an individual level to see if there is gradual learning?"
Response: We agree that our side-bias analyses do not fully rule out subtler strategy differences, and we have added text to the same new Discussion paragraph (p. 26) acknowledging this directly and noting that individual-level, trial-by-trial analyses of learning curves would be needed to test this possibility directly. Importantly, we did interrogate the data to see if performance improved across testing days and if this interacted with trial type (please see below) and neither of these reached statistical significance (reported on pages 16-17 of the RESULTS). Furthermore, the lack of a significant interaction between age x day x trial type also suggests the aged rats did not show a selective improvement in discriminating between the high overlap stimuli over the course of testing.

Major point 4:
"The lack of learning across days is helpful, but trial-type-specific learning could be clarified. The authors report no significant main effect of testing day, suggesting that performance did not generally improve across the three morph testing days. However, it would be useful to clarify whether there was a day × trial type interaction, since this would address whether animals improved specifically on harder morph conditions over time. This matters because learning on specific trial types could further support the idea that animals were acquiring stimulus-response mappings."
Response: We agree this is an important clarification, and apologize for our oversight in neglecting to report this statistic, which was part of the original analysis in the first submission. This has been added to the RESULTS section on pages 16-17. Notably, this interaction and all others did not reach statistical significance. This observation, at least partially, refutes the idea that the aged rats may have improved over training by adopting a different strategy. We note, however, that we cannot completely rule out age-related differences in strategy use.

Major point 5:
"The perirhinal Arc analysis is interesting, but hippocampus seems essential for an MST-framed task. The focus on perirhinal cortex is justified given its role in object and feature processing. However, given the framing around mnemonic similarity and the broader MST literature, I was surprised that hippocampal regions were not examined, particularly dentate gyrus/CA3. A fully functioning perirhinal cortex with dysfunctional hippocampal pattern separation circuitry could still produce mnemonic discrimination impairments under prevailing models. Therefore, the null perirhinal Arc finding should be interpreted cautiously."
Response: This is a fair and important point, and we have revised both the Discussion and our framing of the Arc data accordingly. We have added a new paragraph to the Discussion (pages 24-25) stating explicitly that our Arc analysis was restricted to perirhinal cortex, that hippocampal dentate gyrus/CA3 circuitry is centrally implicated in mnemonic similarity performance (Yassa et al., 2011b; Yassa et al., 2011a; Johnson et al., 2019), and that these regions were not examined here.

We would also like to clarify the scope of our claim. The Arc data were included to test whether perirhinal cortex was differentially recruited as stimulus overlap increased, not to argue that perirhinal cortex was functionally intact in the aged rats. We agree that likely it is not, and the present analysis cannot speak to that question. We therefore now caution explicitly against reading the null perirhinal finding as evidence against the involvement of mnemonic discrimination circuitry more broadly. At most, it suggests that solving the high-overlap condition did not require additional perirhinal recruitment, which raises the possibility of greater reliance on visual association cortices, a possibility the present data cannot test directly.

We hope it is also useful to explain why the catFISH analysis was confined to the perirhinal cortex, for three reasons. First, Arc transcription in the dentate gyrus is prolonged following behavioral experience (Ramirez-Amaya et al., 2013), such that granule cells activated during epoch 1 continue to transcribe Arc whether or not they are active during epoch 2, precluding the epoch-specific cellular resolution on which the catFISH method depends. Second, as we note in our response to this reviewer's second major point, all stimuli had been repeatedly experienced by the time of the Arc session; our previous work indicates that disrupted dentate gyrus/CA3 activity produces mnemonic similarity impairments primarily when stimuli are novel, with the effect diminishing as stimuli become familiar (Johnson et al., 2019), so hippocampal engagement may already have been reduced at this stage of the experiment. Third, we have previously reported that age-related CA3 hyperactivity (Wilson et al., 2005; Yassa et al., 2011b; Robitsek et al., 2015; Thomé et al., 2016; Lee et al., 2022) is most apparent in animals showing mnemonic similarity impairments (Maurer et al., 2017b), which predicts little relationship to behavior in a cohort that was not impaired.

We take the reviewer's broader point, however, that a dissociation between disrupted dentate gyrus/CA3 activity and intact behavior would itself be informative and would bear directly on whether the OCSC task taxes the circuitry that supports mnemonic discrimination abilities. Tissue from these animals has been preserved, and we have made this an explicit direction for future work in the revised Discussion (pages 24-25).

Major point 6:
"Given the possibility that animals may solve the task using rule-based, procedural, or habit-based strategies, future neural analyses could also examine prefrontal cortex and striatal regions."
Response: We agree, and have incorporated this suggestion into the same new Discussion paragraph on trial-uniqueness and strategy (page 27), proposing that future work directly compare perirhinal/hippocampal engagement to prefrontal cortical and striatal activity across the course of training to determine whether, and when, rats shift from a mnemonic to a habit-based strategy on this task.

Reviewer 2
We appreciate the careful read and thoughtful critique of the Reviewer and their note regarding the importance of reporting null results. The primary concern of Reviewer 2 related to the analysis of Arc expression in the perirhinal cortex. The Reviewer suggested that the Arc experiment either be removed from the paper, moved to a supplement, or reworked with limitations noted. We have chosen to do the latter and have revised the Discussion to note limitations of these data as well as cautioning the reader to consider the Arc results as preliminary and hypothesis generating. Additional point-by-point responses are included below.

Main concern 1:
"Firstly, I’m not sure there is enough initial evidence to hypothesize that there would be differences in activity levels of the PRC with different levels of overlap (which is covered in the Discussion). So the lack of effect here is not the same as the lack of effect in the behavioural data where previous results have found a difference, in that case with age."

Response: We appreciate the careful scrutiny of the Arc experiment. In addition to the widely reported finding that perirhinal damage impairs performance in rats and monkeys when there is high feature overlap, but not low (Bartko et al., 2007a, b; Murray et al., 2007), another important justification for this study is that previous functional imaging publications have reported that activation in the perirhinal cortex is higher when participants are discriminating between complex stimuli with high feature overlap compared to when the feature overlap is low (Devlin and Price, 2007; Barense et al., 2010; Ryan et al., 2012). Because Arc activation has been shown to be associated with the BOLD signal (Small et al., 2004; Colon-Perez et al., 2019), it is reasonable to hypothesize that expression of this gene could detect enhanced engagement of a brain region. We were remiss to not include this justification in the original manuscript and have now added it to frame the Arc experiment (page 19). Notably, perirhinal cortical engagement is only observed when the stimuli are a conjunctive of multiple lower features (e.g., shape and texture) or complex stimuli (e.g., faces). The perirhinal cortex is not necessary and not engaged when similarity is only high on a single domain (e.g., size or hue) (Barense et al., 2010; Ryan et al., 2012), which is consistent with the perceptual-mnemonic feature conjunction model of the perirhinal cortex (Bussey et al., 2005; Cowell et al., 2010). We feel that the current null effects (both with behavior and Arc expression) are consistent with the notion that the OCSC task, as implemented here, does not require rats to use conjunctive features to disambiguate the morphed stimuli. This is consistent with the notion that rats of both ages are likely detecting a change in a single dimension to solve the discrimination problem. While a single dimension becomes progressively harder to resolve as the morphs converge, this does not differentiate the discrimination abilities of young and old animals.

Main concern 2:
"The sample size for this experiment is notably quite low, particularly in the aged group. Touchscreen experiments ideally have an n of 16 per group for good statistical power. Here, there are groups as low as 5, which is then divided by sex. In the very least, justification for this low sample size is needed. It should also be considered whether the statistical tests used are valid at such low sample sizes. I wonder if this experiment is better suited for supplemental information as “exploratory analyses” since I don’t think strong conclusions can be drawn from the low sample size.."
Response: We have added explicit caveats to the Discussion (pages 24-26) stating plainly that, given the small sample sizes (young n = 5♀/4♂; aged n = 3♀/2♂) and the complexity of the five-factor LMM, we regard the Arc findings as preliminary and hypothesis-generating rather than conclusive, and we urge caution in interpreting the reported null effects.

Main concern 3:
“Another thing to consider with the Arc experiment is the number of factors going into the main model and whether it would make better scientific or statistical sense to parcellate some of these tests. Particularly the LMM described on pp18-19 has 5 factors, but do the regions and layers need to be directly compared? Or is the more interesting and important analysis whether there are differences within regions? I also wonder if whether analyzing across sex when there are then some groups with ns of 2 or 3 is useful or appropriate or whether for these analyses it would make better statistical sense to collapse across sex.
Response: We agree the five-factor model (Overlap × Region × Layer × Age × Sex) is complex relative to our sample size and have added language to the new Discussion paragraph (p. 24) acknowledging that cell sizes as low as two to three animals per age × sex grouping limit both statistical power and the stability of parameter estimates from this model. We reran the LMM without sex as a factor and the pattern of the results remained the same. Specifically, Arc expression did not differ across stimulus overlap condition (F[1,84] = 0.277, p = 0.60), and there were no significant main effects of age (F[1,12] = 0.001, p = 0.9757), Region (F[1,84] = 2.44, p = 0.12) or Layer (F[1,84] = 0.812, p = 0.37). Group averages and individual animal distributions are shown in Figure 10E (Young rats) and Figure 10F (Aged rats).

Main concern 4:
"While there is some discussion on whether the testing modality (touchscreen vs 3D objects in an arena) influences the outcomes in the MST here vs in previous studies, I think this needs expanded upon. Previous studies have also detected differences in hand-testing vs touchscreen testing (e.g., https://doi.org/10.1523/ENEURO.0120-24.2024) and attributed them to different levels of the cue-context relationship. Touchscreens present the stimuli in a context much more void of “extra-maze” information than tests in standard arenas. Since the results here do not align with previous conventional testing results, the difference likely lies in the context or the stimuli themselves."
Response: We agree that the null age result likely lies in the context or the stimuli themselves and have extensively revised the Discussion to expand on the possible explanations for the lack of an age difference on Page 28, noting that touchscreen chambers present stimuli within a spatially and contextually impoverished environment relative to open arenas. We thank the reviewer for directing us to this literature, which has helped us develop this section considerably. The Sheppard et al. (2024) paper that the Reviewer linked is instructive for our purposes: granular retrosplenial lesions disrupted touchscreen object-location paired-associate learning while sparing spatial working memory and visual discrimination and reversal tested on the same platform, indicating that touchscreen tasks can recruit distinct circuitry depending on whether performance requires integrating information across modalities or processing a single modality alone. This is precisely the distinction we argue is the issue in the present OCSC task.

More broadly, we agree that the testing environment is unlikely to be neutral with respect to the cognitive processes engaged. Procedural and apparatus differences between nominally equivalent recognition memory paradigms have been shown to change the strategies animals adopt and, in turn, which neural systems support performance, with contextual and environmental features of the testing apparatus are among the factors identified (Ameen-Ali et al., 2015). Touchscreen chambers present stimuli within an environment that is spatially and contextually impoverished relative to an open arena, and the stimuli themselves are two-dimensional images rather than three-dimensional objects that can be approached and explored multimodally. This distinction may be consequential: rats trained on 2D "shape" discriminations have been shown to solve them using local luminance cues rather than global shape, acquiring genuinely shape-based solutions only poorly and only when no simpler cue is available (Minini and Jeffery, 2006). Although rodents can under some conditions treat 2D images as equivalent to their 3D counterparts (Cohen et al., 2022), such equivalence cannot be assumed. We have added these considerations to the Discussion as a likely contributor to the discrepancy between the present results and those obtained with 3D object-based rodent MST paradigms, and we note that they converge with our interpretation of the Arc findings. Namely, that the OCSC task as implemented here may be solvable on a single stimulus dimension rather than requiring conjunctive feature processing.

Main concern 5:
"... I believe more discussion is needed on the aspect of what features might be attended to by the rats and how that may affect performance. I was struck by the choice to use images with faces as these contain features, such as eyes, that are naturally more focused upon than other features. In the end, it is unclear whether the aged mice did better than expected or the young mice did worse; it could be that the facial features in the images reduced performance across the board. Discussion on why these ethologically-relevant images were chosen is needed, and perhaps reasons why images from the recent Stark et al MST which are known to work in humans were not used."
Response: This is a thoughtful point we had not adequately addressed. We have added text to the same closing Discussion paragraph (page 28) acknowledging that our bulldog/owl stimuli, chosen for their high initial discriminability and their capacity to be parametrically morphed, may have inadvertently emphasized salient facial features (e.g., eyes) that could be processed differently than other object features, potentially allowing the task to be solved via a small number of highly discriminable cues rather than full configural discrimination, which could mask age-related deficits that depend on fine-grained discrimination. We suggest that future iterations use stimulus sets modeled more directly on those validated in the human MST literature (Stark et al., 2023, already cited), which de-emphasize single, highly salient features.

Minor concerns:
“Spelling and grammar need checked throughout.”
Response: We thank the reviewer for catching these; all five have been corrected with changes indicated in blue font.
Minor concern: Number of sessions per day
"Page 7 and some figures mention the number of days required to reach criterion, but it has not been said how many touchscreen sessions occurred per day. More common notation for this is “Number of Sessions” as it is more specific."
Response: We agree this detail is missing. We have added to the Methods on page 12 that, for all shaping, training and testing, rats participated in one session per day.

Minor concern: Figure 3 S+/S- not defined
"In Figure 3, the S+ and S- are not mentioned in the caption."
Response: Corrected. We have added a sentence to the Figure 3 caption (page 10) defining the correct (S+) and incorrect (S-) response windows shown in the figure.

Minor concern: OCSC/OCSCT abbreviation consistency
"On p9 the abbreviation OCSC is first introduced, and then OCSCT is immediately used, followed by “OCSC task”. If the T in OCSCT is for task, there should be consistency throughout and either only OCSCT or OCSC task be used."
Response: Thank you for catching this. We found this inconsistency (OCSC/OCSCT/OSCST/OSCT) at nine locations throughout the manuscript, including several figure captions, and have standardized all of them to “OCSC task” throughout (changes indicated in blue font throughout).

Minor concern: Non-collapsed overlap conditions
"I'm very curious as to what happens with the data if you do not collapse the overlaps together... Do rats perform any better with one of the stimuli than the other? This might be more my curiosity than a revision necessary in the manuscript!"
Response: We appreciate the reviewer's curiosity. We did look at this initially, and some rats do appear to perform better with one stimulus over another. However, this was not a consistent pattern in the data across all rats. Furthermore, it is not possible to dissociate a stimulus bias from a side bias the way the current task was designed, so for clarity of presentation we collapsed the data.

Minor concern: Normality assumptions
"A lot of the data appear to violate normality assumptions for ANOVAs. Has this been checked and have alternative tests been used or considered?"
Response: As stated in the original Methods, data were screened for normality, homogeneity of variance, and outliers prior to testing, and Greenhouse-Geisser corrections were applied wherever sphericity was violated. We have clarified this further by adding a sentence to the Statistical Analyses paragraph (page 15).

REFERENCES:
Ameen-Ali KE, Easton A, Eacott MJ (2015) Moving beyond standard procedures to assess spontaneous recognition memory. Neuroscience and biobehavioral reviews 53:37–51.
Attalla D, Schatz A, Stumpenhorst K, Winter Y (2024) Cognitive training of mice attenuates age-related decline in associative learning and behavioral flexibility. Front Behav Neurosci 18:1326501.
Barense MD, Henson RN, Lee AC, Graham KS (2010) Medial temporal lobe activity during complex discrimination of faces, objects, and scenes: Effects of viewpoint. Hippocampus 20:389–401.
Bartko SJ, Winters BD, Cowell RA, Saksida LM, Bussey TJ (2007a) Perceptual functions of perirhinal cortex in rats: zero-delay object recognition and simultaneous oddity discriminations. J Neurosci 27:2548–2559.
Bartko SJ, Winters BD, Cowell RA, Saksida LM, Bussey TJ (2007b) Perirhinal cortex resolves feature ambiguity in configural object recognition and perceptual oddity tasks. Learn Mem 14:821–832.
Bussey TJ, Saksida LM, Murray EA (2005) The perceptual-mnemonic/feature conjunction model of perirhinal cortex function. Q J Exp Psychol B 58:269–282.
Cohen SJ, Cinalli DA, Ásgeirsdóttir HN, Hindman B, Barenholtz E, Stackman RW (2022) Mice recognize 3D objects from recalled 2D pictures, support for picture-object equivalence. Scientific Reports 12:4184.
Colon-Perez LM, Turner SM, Lubke KN, Pompilus M, Febo M, Burke SN (2019) Multiscale Imaging Reveals Aberrant Functional Connectome Organization and Elevated Dorsal Striatal. eNeuro 6.
Cowell RA, Bussey TJ, Saksida LM (2010) Components of recognition memory: dissociable cognitive processes or just differences in representational complexity? Hippocampus 20:1245–1262.
Devlin JT, Price CJ (2007) Perirhinal contributions to human visual perception. Curr Biol 17:1484–1488.
Johnson SA, Turner SM, Lubke KN, Cooper TL, Fertal KE, Bizon JL, Maurer AP, Burke SN (2019) Experience-dependent effects of muscimol-induced hippocampal excitation on mnemonic discrimination. Frontiers in systems neuroscience 12:72.
Lee H, Wang Z, Tillekeratne A, Lukish N, Puliyadi V, Zeger S, Gallagher M, Knierim JJ (2022) Loss of functional heterogeneity along the CA3 transverse axis in aging. Curr Biol.
Maurer AP, Johnson SA, Hernandez AR, Reasor J, Cossio DM, Fertal KE, Mizell JM, Lubke KN, Clark BJ, Burke SN (2017) Age-related Changes in Lateral Entorhinal and CA3 Neuron Allocation Predict Poor Performance on Object Discrimination. Front Syst Neurosci 11:49.
Minini L, Jeffery KJ (2006) Do rats use shape to solve "shape discriminations"? Learning & Memory 13:287–297.
Murray EA, Bussey TJ, Saksida LM (2007) Visual perception and memory: a new view of medial temporal lobe function in primates and rodents. Annu Rev Neurosci 30:99–122.
Packard MG, Knowlton BJ (2002) Learning and memory functions of the Basal Ganglia. Annu Rev Neurosci 25:563–593.
Ramirez-Amaya V, Angulo-Perkins A, Chawla MK, Barnes CA, Rosi S (2013) Sustained transcription of the immediate early gene Arc in the dentate gyrus after spatial exploration. J Neurosci 33:1631–1639.
Robitsek J, Ratner MH, Stewart T, Eichenbaum H, Farb DH (2015) Combined administration of levetiracetam and valproic acid attenuates age-related hyperactivity of CA3 place cells, reduces place field area, and increases spatial information content in aged rat hippocampus. Hippocampus 25:1541–1555.
Ryan L, Cardoza JA, Barense MD, Kawa KH, Wallentin-Flores J, Arnold WT, Alexander GE (2012) Age-related impairment in a complex object discrimination task that engages perirhinal cortex. Hippocampus 22:1978–1989.
Sheppard PAS, Oomen CA, Bussey TJ, Saksida LM (2024) The granular retrosplenial cortex is necessary in male rats for object-location associative learning and memory, but not spatial working memory or visual discrimination and reversal, in the touchscreen operant chamber. eNeuro 11:ENEURO.0120–0124.2024.
Small SA, Chawla MK, Buonocore M, Rapp PR, Barnes CA (2004) Imaging correlates of brain function in monkeys and rats isolates a hippocampal subregion differentially vulnerable to aging. Proc Natl Acad Sci U S A 101:7181–7186.
Talpos JC, McTighe SM, Dias R, Saksida LM, Bussey TJ (2010) Trial-unique, delayed nonmatching-to-location (TUNL): a novel, highly hippocampus-dependent automated touchscreen test of location memory and pattern separation. Neurobiol Learn Mem 94:341–352.
Thomé A, Gray DT, Erickson CA, Lipa P, Barnes CA (2016) Memory impairment in aged primates is associated with region-specific network dysfunction. Mol Psychiatry 21:1257–1262.
Wilson IA, Ikonen S, Gallagher M, Eichenbaum H, Tanila H (2005) Age-associated alterarions in place cells are subregion specific. Journal of Neuroscience 25:6877–6886.
Yassa MA, Mattfeld AT, Stark SM, Stark CE (2011a) Age-related memory deficits linked to circuit-specific disruptions in the hippocampus. Proc Natl Acad Sci U S A 108:8873–8878.
Yassa MA, Lacy JW, Stark SM, Albert MS, Gallagher M, Stark CE (2011b) Pattern separation deficits associated with increased hippocampal CA3 and dentate gyrus activity in nondemented older adults. Hippocampus 21:968–979.
Yoder WM, Setlow B, Bizon JL, Smith DW (2014) Characterizing olfactory perceptual similarity using carbon chain discrimination in Fischer 344 rats. Chemical senses 39:323–331.
Yoder WM, Gaynor LS, Burke SN, Setlow B, Smith DW, Bizon JL (2017) Interaction between age and perceptual similarity in olfactory discrimination learning in F344 rats: relationships with spatial learning. Neurobiol Aging 53:122–137.
Zequeira S, Gazarov EA, Güvenli AA, Berthold EC, Senetra AS, Febo M, Hiranita T, McMahon LR, Sharma A, McCurdy CR, Setlow B, Bizon JL (2025) Effects of cannabis smoke and oral Δ9THC on cognition in young adult and aged rats. Psychopharmacology (Berl) 242:835–853.

**Decision Letter (OXFNSC-2026-017.R1)**

**07-Sep-2026**

Dear Dr. Burke,

It is a pleasure to accept your revised manuscript entitled "An Operant-based Touchscreen Morph Discrimination Task Does Not Detect Age-related Mnemonic Similarity Deficits in Rats" in its current form for publication in Oxford Open Neuroscience. Reviewer comments are included at the foot of this letter.

Please note that this journal operates with transparent peer review. This means that  the full peer review history of your article will publish online alongside your article. This includes reviewer comments, editor decision letters, and your author responses.

Next steps
You will receive an email from no-reply@scipris.com within roughly one week. This is your invitation to sign up for an account with SciPris, Oxford University Press’ author portal hosted by Aptara. You will need to create an account if you do not already hold one. Please register or log into your account and follow the online instructions which will guide you through signing your licence and paying the APC. The email and the portal have clearly signposted support options if you need any help during this process.

Please note that SciPris is a completely different system from ScholarOne, so your credentials to submit your manuscript here will not work there. Once you’ve created a SciPris account, you will be able to use it whenever you publish with Oxford Open Neuroscience or any OUP journal. Please note that OUP will only ever request payment for applicable fees be made via SciPris or to an OUP bank account. If you ever have concerns about the legitimacy of a request, please do not hesitate to contact a customer services agent via the SciPris portal or directly via oupsupport@scipris.com.

Thank you for your fine contribution.  On behalf of the Editors of the Oxford Open Neuroscience, we look forward to your continued contributions to the Journal.

Sincerely,
Prof. Alicia Izquierdo
Senior Editor, Oxford Open Neuroscience
aizquie@psych.ucla.edu


Reviewer: 1

Comments to the Author
Thank you for the thorough response. The authors have addressed all my concerns. I think this is a valuable contribution to the field and raises important questions on cross species considerations and why this work is so important. Given that we can't give explicit directions to animal models it always leaves open the question as to how they are solving a task and this is a great example of that.

Reviewer: 2

Comments to the Author
Thank you to the authors for the revisions. I can now recommend the manuscript for publication.

Associate Editor
Comments to the Author:
(There are no comments.)
